# COVID-19 and Mortality, Depression, and Suicide in the Polish Population

**DOI:** 10.3389/fpubh.2022.854028

**Published:** 2022-03-16

**Authors:** Anna Rogalska, Magdalena Syrkiewicz-Świtała

**Affiliations:** Department of Health Economics and Health Management, School of Health Sciences in Bytom, Medical University of Silesia in Katowice, Bytom, Poland

**Keywords:** COVID-19, depression, suicide, mortality, public health

## Abstract

**Aim:**

The study was aimed at assessing the mortality of the population, the occurrence of the phenomenon of depression and suicide before and during the COVID-19 pandemic.

**Methods:**

Data on total mortality in Poland in 2017–2021 came from the report of the Ministry of Health. Data on the number of sick leave due to mental disorders were taken from the report of the ZUS (Social Insurance Institution in Poland). Data on the number of suicides came from police statistics.

**Results:**

Increase in the number of deaths in Poland in 2021 compared to the 2017–2019 average – 26.86%. In 2018–2020, the greatest number of fatal suicides was recorded in the age group – 60–64 years (in 2018 *N* = 565; 10.90%; in 2020 *N* = 524; 10.15%).

**Conclusions:**

In the years 2020-2021, an increase in mortality was observed in Poland compared to the previous years. Moreover, in 2020 there was an increase in sickness absence due to ICD-10 F.32 and an increase in the number of suicide attempts.

## Introduction

There are seven known human coronaviruses (CoVs) that cause respiratory disease (1). The COVID-19 pandemic has affected many aspects of health, has caused serious health and psychosocial problems worldwide, resulting in a fear of infection, feelings of frustration and boredom, insufficient information, and insufficient resources (2) and including population mortality. Death rates are one of the yardsticks used to evaluate health across populations in time. Mortality is easy to measure because of the unambiguous endpoint (3). In turn, excess mortality is the number of deaths from all causes during a crisis above and beyond what we would have expected to see under “normal” conditions. It can be expressed as a rate (the difference between observed and non-crisis mortality rates), or as a total number of excess deaths (4).

Although the general population is susceptible to SARS-CoV-2, many studies have shown that those most at risk of getting SARS-CoV-2 infection are older men and those with underlying diseases (5). Children and infants (especially female infants) were also affected (6). Analyzing the effects of the COVID-19 crisis, it is possible to see differences in individual countries as well as within countries, both in terms of reported cases and related deaths (7). Population density may be one of the putative factors that could influence international variation in the number of COVID-19 cases and deaths. It was in large cities linked by international markets, including business travel and tourism, that were the entry points of the virus. However, it is impossible to establish a clear correlation between the density and the incidence of the disease (8).

The aspect of depression is important in a pandemic situation, due to the documented high incidence of mental illness among people directly or indirectly exposed to life-threatening situations (9). Depression contributes significantly to the global burden of disease and causes high health care expenditure (10). Depression is two to three times more common in people with multiple diseases compared to people with no or no chronic physical disease (11). At worst, depression can lead to suicide. Suicide was the second leading cause of death among people aged 15–29 worldwide in 2015 (12). Worldwide, 703,000 people die as a result of suicide, according to WHO data, and in 2019 more than one in 100 deaths (1.3%) resulted from suicide. The highest suicide rates were in the African region (11.2 per 100,000), followed by Europe (10.5 per 100,000) and Southeast Asia (10.2 per 100,000), exceeding the global average (9.0 per 100,000) in 2019. And the suicide rate in Poland in 2019 was 11.2 per 100,000 (13).

The study was aimed at assessing the mortality of the population, the occurrence of the phenomenon of depression and suicide before and during the COVID-19 pandemic.

## Methods

The research material was collected based on a literature review and available statistical reports. Data on total mortality in Poland in 2017–2021, broken down by weeks, came from the report of the Ministry of Health (https://dane.gov.pl/pl/dataset/1953/liczba-zgonow-zarejestrowanych-w-rejestrze-stanu-cywilnego). Data on the number of sick leave due to mental disorders for the years 2017–2020 were taken from the report of the ZUS (Social Insurance Institution—responsible for insurances in Poland) (https://www.zus.pl/baza-wiedzy/statystyka/opracowania-tematyczne/absencja-chorobowa). For the analysis of sickness absenteeism, we chose Depressive episode, defined in ICD-10 with the code F32, to differentiate it from recurrent depressive disorders, ICD-10 F33. And the data on the number of suicides for 2017–2020 came from police statistics posted on the portal (https://statystyka.policja.pl/st/wybrane-statkieta/zamachy-samobojcze). The obtained results were subjected to basic statistical analyzes.

## Results

### Total Deaths in 2017–2021

In 2017, 404,057 deaths were recorded in Poland, while in 2021 it was 518,693 (an increase of 28.37%).

**Figure 1:**
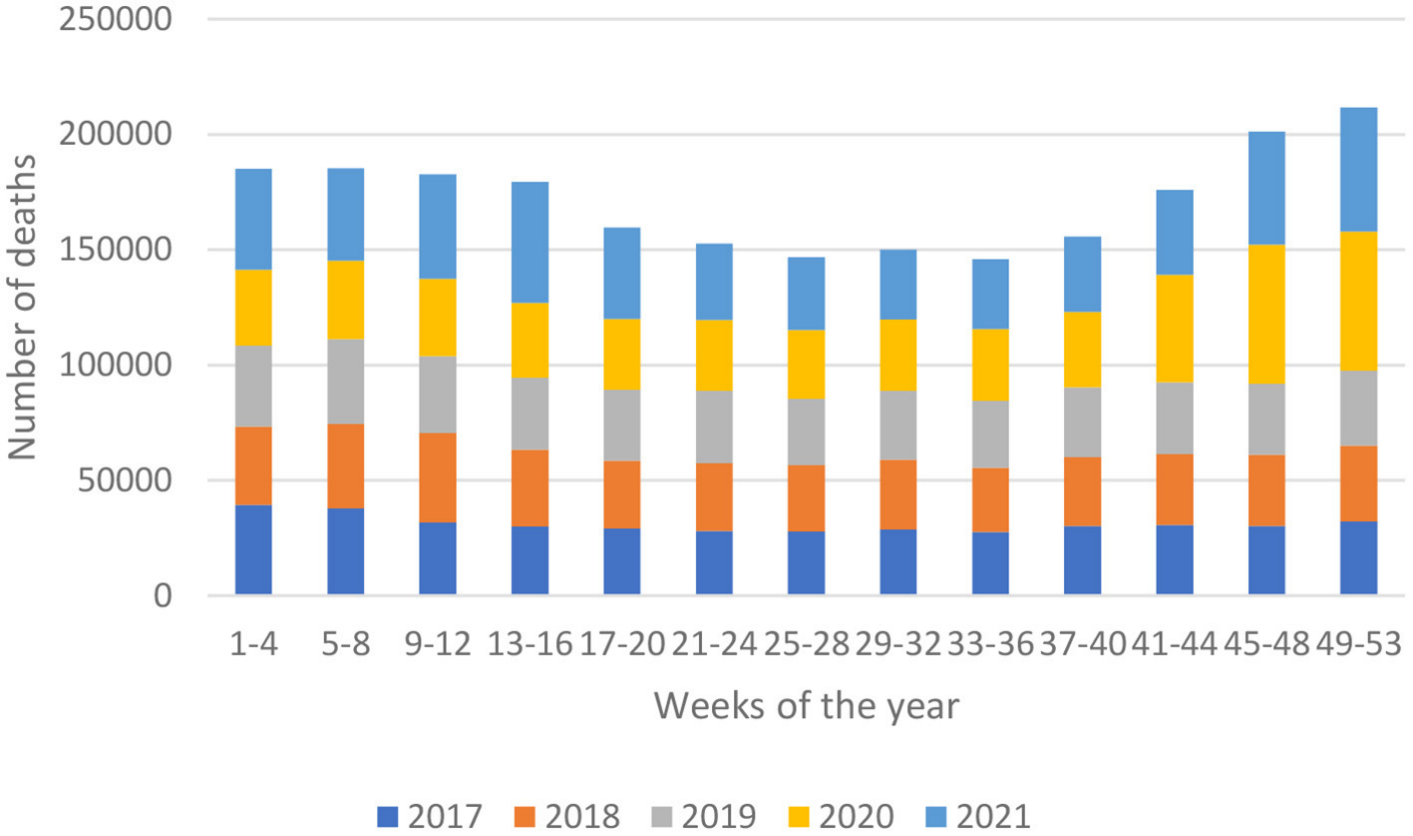
The total number of deaths by weeks in Poland in 2017–2021. Own study based on: https://dane.gov.pl/pl/dataset/1953/liczba-zgonow-zarejestrowanych-w-rejestrze-stanu-cywilnego.

The highest number of deaths in 2021 in Poland was recorded in weeks 49–52 – 53,680 (a decrease of 11.04% compared to 2020). However, in 2020, the highest number of deaths in Poland was recorded in the weeks 45–48 – 60,416 deaths (an increase of 99.31% compared to 2017). However, in 2021 in the same weeks, a decrease in the number of deaths by 18.95% to the number of deaths – 48,968 was observed (Figure 1).

Increase in the number of deaths in Poland in 2020 compared to the 2017-2019 average – 18.97%.

Increase in the number of deaths in Poland in 2021 compared to the 2017-2019 average – 26.86%.

Sickness absenteeism related to a hospital stay of persons insured with ZUS in 2020 accounted for 1.8% of the total number of days of sickness absence and amounted to 4,607.1 thousand. days. Compared to 2019, it was lower by 28.1%.

### Medical Leave Due to Mental Disorders

**Figure 2:**
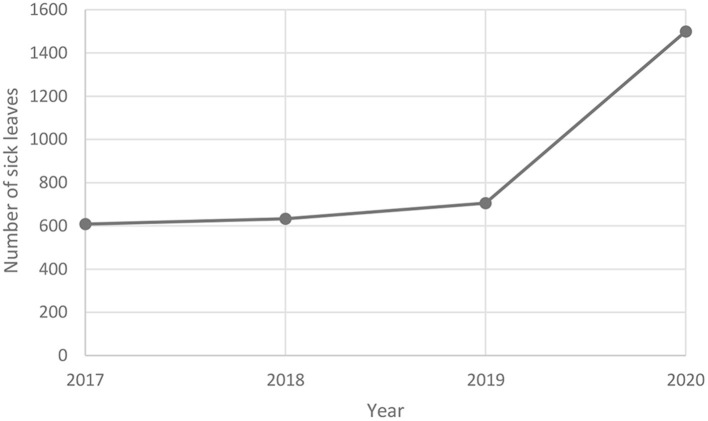
Number of sick leaves due to mental disorders in Poland in 2017–2020. Own study based on: https://www.zus.pl/baza-wiedzy/statystyka/opracowania-tematyczne/absencja-chorobowa.

The number of days of sickness absence due to ICD-10 F.32 in 2020 amounted to 5,197.2 thousand. days (2% all of the disease), which is more than due to COVID-19 – 4,835.2 thousand. days (1.9% of all of the diseases). The number of sickness absences due to ICD-10 F.32 (depressive episode) in 2017-2020 showed an upward trend (Figure 2).

Depressive episode ICD-10 F.32 was known to be the 11th disease entity causing sickness absenteeism due to own disease in men insured with ZUS, and COVID-19 (ICD-10 U07.1) was in the 9th place.

A different situation was observed among women, where the depression episode ICD-10 F.32 (2.4%) was ranked 7th among the disease entities causing the longest sickness absence due to sickness due to sickness in ZUS insured women (2.4%), and COVID-19 (ICD-19) was ranked 7th. 10 U07.1) – 1.8%.

### Fatal Suicides—Age Structure, Gender, Marital Status

In 2017, the highest number of fatal suicides was recorded in the 55-59 age group (N = 603; 11.43%). On the other hand, in 2018-2020, the greatest number of fatal suicides was recorded in the older age group – 60-64 years (in 2018 *N* = 565; 10.90%; in 2019 *N* = 533; 10.14%; in 2020 *N* = 524; 10.15%).

When analyzing the number of fatal suicides among children, the largest number of them was recorded in the age group of 13-18 years (in 2017 *N* = 115; 2.18%; in 2018 *N* = 92; 1.78%; in 2019 *N* = 94; 1.79%; in 2020 *N* = 106; 2.05%).

**Figure 3:**
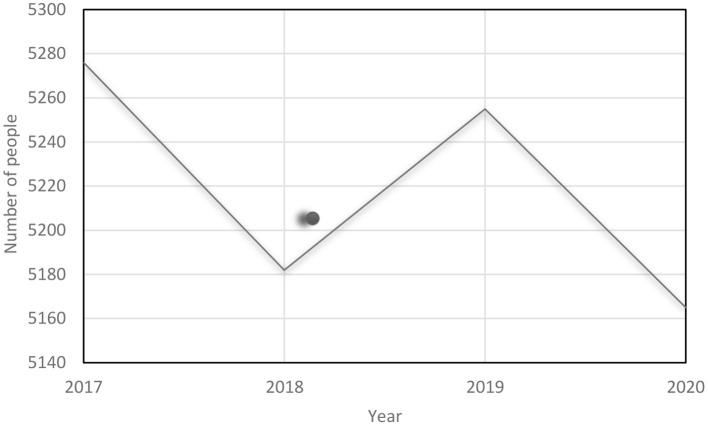
Number of people in fatal suicide attacks in Poland in 2017–2020. Own study based on: https://statystyka.policja.pl/st/wybrane-statystyki/zamachy-samobojcze.

The trend of the total number of fatal suicides in Poland in 2017-2020 is shown in Figure 3.

In terms of gender, men constituted the majority in the entire analyzed period (in 2017 *N* = 4,524; 85.76%; in 2018 *N* = 4,471; 86.28%; in 2019 *N* = 4,497; 85.58%; in 2020 *N* = 4,386; 84.92%).

In relation to marital status, married / married status dominated in each of the 4 years (in 2017 *N* = 2013; 38.15%; in 2018 *N* = 1,999; 38.58%; in 2019 *N* = 1,973; 37.54%; in 2020 *N* = 1,896, 36.70%).

### Total Suicide—Age Structure, Gender, Marital Status

The total number of suicide attacks shows an upward trend: in 2017 *N* = 11,139 (11.2 per 100,000); in 2018 *N* = 11,167 (11.2 per 100,000); in 2019 *N* = 11961 (11.2 per 100,000); in 2020 *N* = 12,013 (12.0 per 100,000). In 2020, an increase of 7.85% was observed compared to 2017. In the total number of suicide attacks in 2017–2019, people in the 30-34 age group were the largest (in 2017 *N* = 1,263; 11.33%; in 2018 *N* = 1,226; 10.98%; in 2019 *N* = 1,363; 11.40%). However, in 2020, the largest group were people aged 35–39 (N = 1,390; 11.57%). Among children, the largest number of suicide attacks was recorded in the age group of 13–18 years (in 2017 *N* = 702; 6.30%; in 2018 *N* = 746; 6.68%; in 2019 *N* = 905; 7.57%; in 2020 *N* = 814; 6.78%). When analyzing suicide attacks by gender, the largest number was recorded in the group of men (in 2017 *N* = 8,515; 76.44%; in 2018 *N* = 8364; 74.90; in 2019 *N* = 8782; 73.42; in 2020 *N* = 8796; 73.22%). In terms of marital status, most people were unmarried / single (in 2017 *N* = 4,315; 38.73%; in 2018 *N* = 4,298; 38.49%; in 2019 *N* = 4,798; 40.11%; in 2020 *N* = 4,882; 40.63%).

## Discussion

Due to the rapid spread of the COVID-19 pandemic, the virus has led to considerable mortality and morbidity worldwide (14). According to WHO data, as of July 7, 2021, 184.324 026 confirmed cases of COVID-19, including 3,992,680 deaths, reported to WHO, were reported worldwide. Of which the largest number of deaths is in North and South America than in Europe and Southeast Asia (15). The largest excess of deaths was recorded in the United States, Italy, England and Wales, Spain, and Poland (16). Although Poland, like the Czech Republic, Slovakia, Hungary, Denmark, Finland, Bulgaria, Australia, was one of the countries that avoided a detectable increase in mortality from any cause in the first wave of the COVID-19 epidemic (late-May 2020) (17), it turned into in the second half of 2020. This study shows that Poland is one of the countries where excessive mortality was observed during the COVID-19 pandemic comparing the number of deaths in 2019 to 2020 (17). Mortality influence of the COVID-19 pandemic varies from country to country due to the sociodemographic characteristics of the population, the extent and timing of the epidemic and the response, the overall health status of the population. Moreover, differentiated mortality in individual countries is influenced by factors such as the resilience and agility of the health and social care system and the effectiveness of social and economic safety nets that support those in need (17). In our study, we presented the% increase in mortality in 2021 compared to the 2017-2019 average – 26.86%.

De Larochelambert et al. showed that higher Covid-19 mortality rates are mainly found in countries where life expectancy is longer and there is a recent slowdown in this progression (18). Most of these advanced and aging societies are situated latitudinally at 25.

It has been shown that population density related to poverty, poor housing, and limited access to healthcare is a problem. In the United States, factors that may be “responsible” for COVID-19 deaths include household crowding, poverty, socio-economic segregation, and participation in the workforce (19, 20). Factors that are associated with COVID-19 mortality, such as old age, ethnicity, obesity, hypertension, cardiovascular disease, and diabetes. Not only did virus deaths contribute to the increased mortality, but also deaths from other causes, driven by complex factors. The number of SARS deaths depends on the capacity of the healthcare system, which in the model is simulated by the number of available respiratory ventilators or intensive care beds (21). The course of the pandemic will depend on early implementation, mass diagnosis and contact tracing/isolation, and physical distance (22). In addition to the impact of the COVID-19 pandemic on the physical condition of society, effects related to the impact on the mental state and wellbeing of individuals have also been observed (2). Fiorillo et al. argue that the psychosocial consequences of the COVID-19 pandemic can be particularly severe for at least four groups of people: (a) those who have had direct or indirect contact with the virus; (b) individuals who are already susceptible to biological or psychosocial stressors; c) healthcare professionals; and (d) people who follow news through multiple media channels (23).

The increase in the number of deaths at the end of 2020 was caused not only by COVID-19 but also due to other diseases caused by limited access to healthcare, as well as the high burden on the health care system (24). In turn, Grabowski et al. suggest the following reasons: underdiagnosis of COVID-19 cases, as well diagnosis of COVID-19 in hospitalized patients had a major impact on operations, including quarantining staff and even closing entire wards, limiting access to health services for the most seriously ill (25). We hypothesize that one of the reasons could also be the patients' fear of falling ill with COVID-19, which consequently delayed their reporting to doctors. Another reason could also be the limited access to specialist doctors during the COVID-19 pandemic, which in turn resulted in increased use of ambulance services, thus making access difficult for people in a life-threatening condition. In addition, some ambulances waited for a long time (waiting for the COVID-19 test result) in front of the hospital before the patient was admitted.

One of the countries that also experienced excess mortality in 2020 was Italy, where inconsistencies in regional health systems and their delayed response in some areas were identified as the causes of this state of affairs (26). In Portugal, however, Vieira et suggested causes of excess mortality such as: some patients may have died from COVID-19; they may have died at home or in a long-term care facility without being tested for COVID-19; other seriously ill patients may not have sought hospital care for fear of infection or have sought hospital care, but have not received all the attention they need as the workforce and equipment were dedicated to caring for COVID-19 patients (27). Similarly, researchers from Belgium, where there was also excess mortality in 2020. Excessive mortality during the first wave of the COVID-19 epidemic suggested that it could be caused by a delay in the treatment of both acute and chronic pathologies, as well as difficulties in accessing care during the blockade and with patients who hesitate to seek medical attention for fear of contracting the virus (28).

The second aspect we examined was the impact of the COVID-19 pandemic on the number of sick leaves due to mental disorders. In the conducted study, there was a significant increase, by 25.3% in the number of sick leave for this reason. An upward trend was also observed by Robillard et al., where nearly half of a large sample of Canadians who had reported no previous psychiatric history tested positive on standard clinical tools for clinical depression during the COVID-19 pandemic (29). Emotional reactions caused by Covid-19 Pandemia can be more significant among groups susceptible to threats, such as people with previously existing mental diseases (30). A group of enhanced risk on mental disorders may be protecting employees (31, 32), older adults (33), the homeless (34). It has been proven that the explosions of infectious diseases such as COVID-19 are associated with mental anxiety and symptoms of mental illness (35). Researchers suggest that the pandemic has led to many problems such as prolonged anxiety and stress, increased physical distances between people, loneliness and isolation, and job loss (36), which may be the reason for the increase in the number of sick leaves due to mental disorders. As demonstrated in their research by Sakakibara et al. diagnosis, many previous sick leave episodes, and employee rank are predictors of the duration of sick leave due to mental disorders (37).

There are concerns in scientific publications about an increase in the number of suicides as one of the consequences of the COVID-19 pandemic (38, 39). Based on police data in Poland, no increase in the number of fatal suicides in 2020 was shown. No impact of the COVID-19 pandemic on the rise in the number of suicides was observed in Korea, where the number of suicides even decreased by 6.9% in the first eight months of 2020 compared to the same period in 2019 (40). When analyzing demographic factors in Poland, more men who committed suicide ended with death, which is similar to the situation in other countries such as India (80.8%) (41) and Australia (42), as well as in the European Union countries (77%) (43). Research suggests that> 90% of suicide victims have mental disorders (44). In response to the constraints associated with isolation and compliance with barriers, it may be a good strategy to use e-health tools, also for suicide prevention (45). The limitation of the study is the lack of an assessment of the long-term impact of the COVID-19 pandemic. More research is needed in this area. It seems important to take appropriate corrective actions to stop the upward trend in the number of mental disorders. It would be useful to introduce evidence-based activities in the field of psychological education and coping with stress at the school level and to increase access to psychotherapeutic services as part of outpatient care within the public health system. Examples of specific solutions include the following strategies: developing teams of specialists qualified to solve emotional problems (46), developing online resources for mental health education (47), training community staff in basic aspects of psychiatric care (46).

## Conclusions

In Poland, during the COVID-19 pandemic in the years 2020–2021, a higher total number of deaths was observed compared to the average number of deaths in 2017–2019. It seems important to take appropriate corrective actions to stop the upward trend in the number of deaths. Moreover, there was an increase in sickness absence due to ICD-10 F.32 (depressive episode) in 2020. In 2020, there was an increase in suicide attempts compared to 2017.

## Data Availability Statement

The original contributions presented in the study are included in the article/supplementary material, further inquiries can be directed to the corresponding author/s.

## Author Contributions

AR conceived the study and prepared draft of the paper. MS-Ś and AR contributed to paper preparation and study and provided new information necessary to revise the paper. All authors contributed to the article and approved the submitted version.

## Conflict of Interest

The authors declare that the research was conducted in the absence of any commercial or financial relationships that could be construed as a potential conflict of interest.

## Publisher's Note

All claims expressed in this article are solely those of the authors and do not necessarily represent those of their affiliated organizations, or those of the publisher, the editors and the reviewers. Any product that may be evaluated in this article, or claim that may be made by its manufacturer, is not guaranteed or endorsed by the publisher.

## References

[1] Menicagli R Limodio M. COVID-19 solution. Int J Prev Med. (2020) 11:73. 10.4103/ijpvm.IJPVM_227_20 32742617 PMC7373088

[2] Brooks SK Webster RK Smith LE Woodland L Wessely S Greenberg N. The psychological impact of quarantine and how to reduce it: rapid review of the evidence. Lancet (London, England). (2020) 395:912–20. 10.1016/S0140-6736(20)30460-8 32112714 PMC7158942

[3] Bonneux L. How to measure the burden of mortality? J Epidemiol Commun Health. (2002) 56:128–31. 10.1136/jech.56.2.128 11812812 PMC1732067

[4] Checchi F Roberts L. Interpreting and using mortality data in humanitarian emergencies. A primer for non-epidemiologists. Humanitarian Pract Netw. (2005) 5:1–38.

[5] Guo G Ye L Pan K Chen Y Xing D Yan K. New Insights of Emerging SARS-CoV-2: epidemiology, etiology, clinical features, clinical treatment, and prevention. Front Cell Dev Biol. (2020) 8:410. 10.3389/fcell.2020.00410 32574318 PMC7256189

[6] Liu W Zhang Q Chen J Xiang R Song H Shu S. Detection of Covid-19 in children in Early January 2020 in Wuhan, China. N Engl J Med. (2020) 382:1370–1. 10.1056/NEJMc2003717 32163697 PMC7121643

[7] (COVID-19) OPRtC. The territorial impact of COVID-19: Managing the crisis across levels of government 2020 Available from: http://www.oecd.org/coronavirus/en/

[8] OECD. Cities policy responses 2020. Available online at: http://www.oecd.org/coronavirus/policy-responses/cities-policy-responses-fd1053ff/ (accessed January 25, 2021).

[9] Chen Q Liang M Li Y Guo J Fei D Wang L. Mental health care for medical staff in China during the COVID-19 outbreak. Lancet Psychiatry. (2020) 7:e15–e6. 10.1016/S2215-0366(20)30078-X 32085839 PMC7129426

[10] Konig H Konig HH Konnopka A. The excess costs of depression: a systematic review and meta-analysis. Epidemiol Psychiatr Sci. (2019) 29:e30. 10.1017/S2045796019000180 30947759 PMC8061284

[11] Read JR Sharpe L Modini M Dear BF. Multimorbidity and depression: A systematic review and meta-analysis. J Affect Disord. (2017) 221:36–46. 10.1016/j.jad.2017.06.009 28628766

[12] WHO. Depression and Other Common Mental Disorders. Global Health Estimates. 2017 Geneva (2017).

[13] WHO. Suicide worldwide in 2019. Global Health Estimates (2021).

[14] Rui JR Yang K Chen J. Information sources, risk perception, and efficacy appraisal's prediction of engagement in protective behaviors against COVID-19 in China: repeated cross-sectional survey. JMIR Hum Factors. (2021) 8:e23232. 10.2196/23232 33338027 PMC7806274

[15] WHO. COVID-19 2021 Available from: https://covid19.who.int/

[16] Islam N Shkolnikov VM Acosta RJ Klimkin I Kawachi I Irizarry RA. Excess deaths associated with covid-19 pandemic in 2020: age and sex disaggregated time series analysis in 29 high income countries. BMJ. (2021) 373:n1137. 10.1136/bmj.n1137 34011491 PMC8132017

[17] Kontis V Bennett JE Rashid T Parks RM Pearson-Stuttard J Guillot M. Magnitude, demographics and dynamics of the effect of the first wave of the COVID-19 pandemic on all-cause mortality in 21 industrialized countries. Nat Med. (2020) 26:1919–28. 10.1038/s41591-020-1112-0 33057181 PMC7615092

[18] De Larochelambert Q Marc A Antero J Le Bourg E Toussaint JF. Covid-19 mortality: a matter of vulnerability among nations facing limited margins of adaptation. Front Public Health. (2020) 8:604339. 10.3389/fpubh.2020.604339 33330343 PMC7710830

[19] Basset M. Just Because You Can Afford to Leave the City, Doesn't Mean You Should. (2020) Available online at: https://www.nytimes.com/2020/05/15/opinion/sunday/coronavirus-cities-density.html

[20] Singu S Acharya A Challagundla K Byrareddy SN. Impact of social determinants of health on the emerging COVID-19 pandemic in the United States. Front Public Health. (2020) 8:406. 10.3389/fpubh.2020.00406 32793544 PMC7385373

[21] Burda Z. Modeling excess mortality in Covid-19-Like epidemics. Entropy (Basel). (2020) 22:11. 10.3390/e22111236 33287004 PMC7712842

[22] Abu-Raya B Migliori GB O'Ryan M Edwards K Torres A Alffenaar JW. Coronavirus Disease-19: an interim evidence synthesis of the world association for infectious diseases and immunological disorders (Waidid). Front Med (Lausanne). (2020) 7:572485. 10.3389/fmed.2020.572485 33195319 PMC7662576

[23] Fiorillo A Gorwood P. The consequences of the COVID-19 pandemic on mental health and implications for clinical practice. Eur Psychiatry. (2020) 63:e32. 10.1192/j.eurpsy.2020.35 32234102 PMC7156565

[24] Czerwiński A. Nadmierna śmiertelność w Polsce w 2020 r., Working Paper. Polski Instytut Ekonomiczny, Warszawa (2021).

[25] Grabowski J Witkowska N Bidzan L. Letter to the editor: Excess all-cause mortality during second wave of COVID-19—the Polish perspective. Euro Surveill. (2021) 26:7. 10.2807/1560-7917.ES.2021.26.7.2100117 33602384 PMC7897912

[26] Nardini S Sanguinetti CM De Benedetto F Baccarani C Del Donno M Polverino M. SARS-CoV-2 pandemic in Italy: ethical and organizational considerations. Multidiscip Respir Med. (2020) 15:672. 10.4081/mrm.2020.672 32499910 PMC7256627

[27] Vieira A Peixoto VR Aguiar P Abrantes A. Rapid estimation of excess mortality during the covid-19 pandemic in portugal -beyond reported Deaths. J Epidemiol Glob Health. (2020) 10:209–13. 10.2991/jegh.k.200628.001 32954711 PMC7509098

[28] Bustos Sierra N Bossuyt N Braeye T Leroy M Moyersoen I Peeters I. All-cause mortality supports the COVID-19 mortality in Belgium and comparison with major fatal events of the last century. Arch Public Health. (2020) 78:117. 10.1186/s13690-020-00496-x 33292536 PMC7662738

[29] Robillard R Daros AR Phillips JL Porteous M Saad M Pennestri MH. Emerging New Psychiatric Symptoms and the Worsening of Pre-existing Mental Disorders during the COVID-19 Pandemic: A Canadian Multisite Study: Nouveaux symptomes psychiatriques emergents et deterioration des troubles mentaux preexistants durant la pandemie de la COVID-19: une etude canadienne multisite. Can J Psychiatry. (2021) 2021:706743720986786. 10.1177/0706743720986786 33464115 PMC8504288

[30] Yao H Chen JH Xu YF. Patients with mental health disorders in the COVID-19 epidemic. Lancet Psychiatry. (2020) 7:e21. 10.1016/S2215-0366(20)30090-0 32199510 PMC7269717

[31] Ornell F Halpern SC Kessler FHP Narvaez JCM. The impact of the COVID-19 pandemic on the mental health of healthcare professionals. Cad Saude Publica. (2020) 36:e00063520. 10.1590/0102-311x00063520 32374807

[32] Tsamakis K Rizos E Manolis AJ Chaidou S Kympouropoulos S Spartalis E. COVID-19 pandemic and its impact on mental health of healthcare professionals. Exp Ther Med. (2020) 19:3451–3. 10.3892/etm.2020.8646 32346406 PMC7185082

[33] Yang Y Li W Zhang Q Zhang L Cheung T Xiang YT. Mental health services for older adults in China during the COVID-19 outbreak. Lancet Psychiatry. (2020) 7:e19. 10.1016/S2215-0366(20)30079-1 32085843 PMC7128970

[34] Tsai J Wilson M. COVID-19: a potential public health problem for homeless populations. Lancet Public Health. (2020) 5:e186–e7. 10.1016/S2468-2667(20)30053-0 32171054 PMC7104053

[35] Bao Y Sun Y Meng S Shi J Lu L. 2019-nCoV epidemic: address mental health care to empower society. Lancet (London, England). (2020) 395(10224):e37-e8. 10.1016/S0140-6736(20)30309-3 32043982 PMC7133594

[36] Fushimi M. The importance of studying the increase in suicides and gender differences during the COVID-19 pandemic. QJM. (2021) 21:130. 10.1093/qjmed/hcab130 33964169 PMC8135995

[37] Sakakibara S Sado M Ninomiya A Arai M Takahashi S Ishihara C. Predictive factors of the duration of sick leave due to mental disorders. Int J Ment Health Syst. (2019) 13:19. 10.1186/s13033-019-0279-6 30976299 PMC6441213

[38] Gunnell D Appleby L Arensman E Hawton K John A Kapur N. Suicide risk and prevention during the COVID-19 pandemic. Lancet Psychiatry. (2020) 7:468–71. 10.1016/S2215-0366(20)30171-1 32330430 PMC7173821

[39] Klomek AB. Suicide prevention during the COVID-19 outbreak. Lancet Psychiatry. (2020) 7:390. 10.1016/S2215-0366(20)30142-5 32353271 PMC7185940

[40] Kim AM. The short-term impact of the COVID-19 outbreak on suicides in Korea. Psychiatry Res. (2021) 295:113632. 10.1016/j.psychres.2020.113632 33338860 PMC7718107

[41] Panigrahi M Pattnaik JI Padhy SK Menon V Patra S Rina K. COVID-19 and suicides in India: A pilot study of reports in the media and scientific literature. Asian J Psychiatr. (2021) 57:102560. 10.1016/j.ajp.2021.102560 33465521 PMC7804380

[42] Martinez-Rives NL Dhungel B Martin P Gilmour S. Method-specific suicide mortality trends in australian men from 1978 to 2017. Int J Environ Res Public Health. (2021) 18(9). 10.3390/ijerph18094557 33923084 PMC8123328

[43] Eurostat. Deaths due to intentional self-harm. (2016). Available online at: https://ec.europa.eu/eurostat/en/web/products-eurostat-news/-/edn-20190909-1.

[44] Sher L. Resilience as a focus of suicide research and prevention. Acta Psychiatr Scand. (2019) 140:169–80. 10.1111/acps.13059 31150102

[45] Conejero I Berrouiguet S Ducasse D Leboyer M Jardon V Olie E. [Suicidal behavior in light of COVID-19 outbreak: clinical challenges and treatment perspectives]. Encephale. (2020) 46(3S):S66–S72. 10.1016/j.encep.2020.05.001 32471707 PMC7205618

[46] Duan L Zhu G. Psychological interventions for people affected by the COVID-19 epidemic. Lancet Psychiatry. (2020) 7:300–2. 10.1016/S2215-0366(20)30073-0 32085840 PMC7128328

[47] Liu S Yang L Zhang C Xiang YT Liu Z Hu S. Online mental health services in China during the COVID-19 outbreak. Lancet Psychiatry. (2020) 7:e17–e8. 10.1016/S2215-0366(20)30077-8 32085841 PMC7129099

